# A Comparison of Microsatellites in Phytopathogenic *Aspergillus* Species in Order to Develop Markers for the Assessment of Genetic Diversity among Its Isolates

**DOI:** 10.3389/fmicb.2017.01774

**Published:** 2017-09-20

**Authors:** Sahil Mahfooz, Satyendra P. Singh, Nishtha Mishra, Aradhana Mishra

**Affiliations:** Division of Plant Microbe Interaction, CSIR-National Botanical Research Institute Lucknow, India

**Keywords:** microsatellite, comparative genomics, *Aspergillus*, polymorphism, motif conservation

## Abstract

The occurrence of Microsatellites (SSRs) has been witnessed in most of the fungal genomes however its abundance varies across species. In the present study, we analyzed the frequency of SSRs in the whole genome and transcripts of two phyto-pathogenic (*Aspergillus niger* and *Aspergillus terreus*) and compared them with two non-pathogenic (*Aspergillus nidulans* and *Aspergillus oryzae*) *Aspergillus*. Higher relative abundance and relative density of SSRs were observed in the whole genome and transcript sequences of the pathogenic *Aspergillus* when compared to the non-pathogenic. The relative abundance and density of SSRs were positively correlated with the G+C content of transcripts. Among the different classes of SSR, the percentage of tetra-nucleotide SSRs were maximum in *A. niger* (36.7%) and *A. oryzae* (35.9%) whereas *A. nidulans* and *A. terreus* preferred tri-nucleotide SSRs (38.2 and 42.1%) in whole genome sequences. In transcripts, tri-nucleotide SSRs were the most abundant whereas di-nucleotide SSRs were the least favored. Motif conservation study among the transcripts revealed conservation of only 27% motif within *Aspergillus* species. Furthermore, a similar relationship among the Ascomycetes was obtained on the basis of motif conservation and conserved genes (rDNA). To analyze the diversity present within the Indian isolates of *Aspergillus*, primers were successfully designed for 692 motifs in *A. niger* and *A. terreus* of which 20 were selected for diversity analysis. Among all the markers amplified, 10 markers (83.3%) were polymorphic, whereas remaining two markers (16.6%) were monomorphic. *Ten* polymorphic markers acquired in this investigation showed the utility of recently created SSR markers in the assessment of genetic diversity among various isolates of *Aspergillus*.

## Introduction

Species of the filamentous fungal genus *Aspergillus* displays a wide variation and are of high significance to people (Gibbons and Rokas, 2013). Among many species, *Aspergillus niger* is a soil saprobe and widely used in fermentation industry for the production of enzymes and organic acids (Pel et al., 2007). It is also responsible for the degradation of various organic substances including fruits, vegetables, beans, and cereals (Baker, 2006). In India, *A. niger* has been reported for causing necrotic leaf spot disease in *Zingiber officinale* (Pawar et al., 2008). *Aspergillus terreus* has been widely known for the production of Lovastatin, a polyketide derivative used for lowering cholesterol. This fungus is having a reputation for being the third most common reason of invasive aspergillosis in humans. Mycotoxins produced by it causes food spoilage in several cereals and nuts grown in tropical and subtropical climates (Kuck et al., 2014). A recent report suggests its involvement in causing foliar blight of potato in India (Louis et al., 2013).

The genome of this genus is widely studied, curated, and annotated under the *Aspergillus* Genome Database (AspGD) where the whole genome sequences of several members of this genus are publically available (Arnaud et al., 2012). With the availability of the genome sequences from various species along with the development of many bioinformatics tools, it is now possible for the researchers to use the sequence information for various purposes. These tools provide an ease for developing high throughput *in-silico* methods for the better understanding and characterization of the *Aspergillus* population and developing markers for their identification.

Though the molecular marker technologies such as random amplified polymorphic DNA (RAPD), restriction fragment length polymorphism (RFLP), amplified fragment length polymorphism (AFLP), and inter simple sequence repeats (ISSR) were utilized in *Aspergillus* yet they tend to exhibit reproducibility issues and were insufficient in evaluating intra species variation (Semighini et al., 2001; Baddley et al., 2003; Schmidt et al., 2004; Kermani et al., 2016).

Microsatellites or Simple Sequence Repeats (SSRs) are the parts of the genome comprising a succession of repeats of a given series of nucleotides having lengths from one to six bases. A large instability in the number of repeats is witnessed due to the addition or deletion of repeated units prompting variation in the number of motifs (Gur-Arie et al., 2000; Lim et al., 2004; Olango et al., 2015). Microsatellites can be found in the protein-coding (Li et al., 2004; Garnica et al., 2006; Lawson and Zhang, 2006; Mahfooz et al., 2012a) and non-coding regions of the genome (Kim et al., 2008; Araujo et al., 2012). Microsatellite loci show extensive length polymorphism, and hence they are widely used for DNA fingerprinting and diversity studies in bacteria (Mrazek et al., 2007; Guo and Mrazek, 2008), fungi (Kim et al., 2008; Araujo et al., 2012; Mahfooz et al., 2012a,b), plants (Datta et al., 2010; Yu et al., 2017), and human (Subramanian et al., 2003; Shin et al., 2017). The utility of microsatellites as a molecular marker is well-known, however, its presence and absence in a particular species are of great functional and evolutionary significance (Gibbons and Rokas, 2009; Mahfooz et al., 2015, 2016).

In spite of the fact that the genome sequences of *Aspergillus* species are freely available, SSRs were analyzed only in intergenic sequences (Gibbons and Rokas, 2009), leaving the remaining portion of the genome unexplored. In the present study we wanted to address (1) whether there is any difference between the frequency and distribution among phytopathogenic SSRs (2) the strength of phylogenetic signal at the species level (3) the level of motif conservation among *Aspergillus* species, and (4) the level of motif conservation among Ascomycota. We observed that the frequency of SSRs was higher in the phyto-pathogenic SSRs as compared to its non-pathogenic neighbor. Primers were designed and validated for their ability to revealed polymorphism in Indian isolates of *Aspergillus* isolated from different hosts.

## Materials and methods

### SSR mining

The entire genome sequence of *A. niger, A. terreus, A. nidulans*, and *A. oryzae* were downloaded from the Aspergillus Genome Database (AspGD; http://www.aspgd.org; Arnaud et al., 2012). The scanning of microsatellites was performed using online tool WebSat (Martins et al., 2009). Repeats more than 12 bp were considered as SSRs, which means that only more than six occurrences of a di-nucleotide repeats, four occurrences of a tri-nucleotide repeats, three occurrence of a tetra-, penta-, and hexa-nucleotide repeats will be considered as SSRs. All SSRs were analyzed for their frequency of occurrence, density, and relative abundance. Density was calculated by dividing the number of base pairs contributed by each SSR by total length analyzed (Mb). Relative abundance was calculated as the number of SSRs per Mb of a sequence. While scanning di- to hexa-nucleotide SSRs, combinations involving runs of the same nucleotide were considered. In the current analysis, each SSR was considered as unique.

For a superior comparison of the developmental relationship among the *Aspergillus* species, sharing of repeats was analyzed within the transcribed sequences. As previously reported (Mahfooz et al., 2015), motif sharing within the transcripts of *Aspergillus* species was investigated manually in Microsoft Excel workbook, 2007. Every motif obtained in the transcripts of each species was placed in Microsoft Excel sheet and searched for its counterpart in the transcripts of remaining species. If motif was available in all the four transcripts, it was deemed as common. Thus, the motifs shared between two and three transcript sequences were also analyzed. The motif which did not have any match was considered as novel. PRIMER 3 online software program (frodo.wi.mit.edu/) was used to design primers complementary to the flanking regions of microsatellites. We expected the primers to be 18–24 bp in length, with annealing temperature in the range of 54–62°C, and product lengths between 150 and 400 bp. A total of 2,169 primers were designed from the four *Aspergillus* species (Supplementary Table 1). Online software GC content calculator (http://www.sciencebuddies.org/science-fair-ojects/project_ideas/Genom_GC_Calcu-lator.shtml) was used to calculate the G+C content of the genomes and transcripts. Pearson correlation coefficient was calculated using SPSS package (SPSS V16.0, SPSS Inc., Chicago, IL, USA).

### Fungal isolates

A total of 23 different *Aspergillus* isolates representing 11 each from *A. niger* and *A. terreus* along with one from *A. nidulans* was obtained from Department of Plant Pathology, Indian Agricultural Research Institute, Pusa, New Delhi, India (Supplementary Table 2). These isolates represent the diverse agro-climatic zones of India.

### DNA isolation and SSR amplification

Total genomic DNA from 23 *Aspergillus* isolates was extracted using HiPurA™ Fungal DNA Purification Kit (HIMEDIA, India). The PCR was performed in 10.0 μl reaction volume containing PCR buffer (10 mM Tris-HCl pH 9.0, 1.5 mM MgCl_2_, 50 mM KCl, 0.01% gelatine), 200 μM each of dNTP (Merck), 0.2 U of Taq DNA polymerase (Merck), 10 pM each of forward and reverse primers, and 10 ng of genomic DNA was used as template in each PCR reaction. PCR program was as follows: after initial denaturation at 95°C for 3 min, five touch-down PCR cycles comprising of 94°C for 20 s, 60/55°C for 20 s, and 72°C for 30 s were performed. These cycles were subsequently followed by 40 cycles of denaturation at 94°C for 20 s with a constant annealing temperature of 54–60°C (depending on primer) for 20 s, and extension at 72°C for 20 s, and a final extension at 72°C for 20 min. All PCR amplicons were resolved by electrophoresis on 2% agarose gel to identify the informative SSR loci across all the isolates.

### Statistical analysis

The amplification information produced by SSR primers was examined using SIMQUAL (Nei and Li, 1979) to create a Jaccard's similarity coefficient utilizing NTSYS-PC, programming version 2.1. These similarity coefficients were utilized to develop a dendrogram delineating hereditary relationship among the *Aspergillus* isolates by utilizing the Unweighted Paired Group Method of Arithmetic Averages (UPGMA). The allelic differences or polymorphism information content (PIC) was measured as described by Botstein et al. (1980). PIC is characterized as the likelihood that two randomly picked duplicates of a gene will represent different alleles within a population. The PIC value was calculated with the equation as follows:

$${\text{PIC}}_{i}=1-\sum_{j=1}^{n}{\text{Pij}}^{2}$$

where P_i_ represents the frequency of the j^th^ pattern for marker i, and summation extends over n patterns. A phylogenetic tree of the 18S rRNA gene was also constructed using Clustal W programme in the MEGA 5.2 software (Treangen and Messeguer, 2006) using the neighbor-joining algorithm with bootstrap analysis for 1,000 replicates.

## Results

### Relative abundance and density of SSRs in the genomic sequences of *Aspergillus* spp.

The maximum frequency of SSRs in whole genome sequences was identified in the genome of *A. niger* (8,896) which was much higher when compared with *A. oryzae* (5,226), *A. terreus* (4,823), and *A. nidulans* (2,919). The data suggested that *A. oryzae* which had the largest genome size contains the second highest frequency of SSRs whereas *A. niger* which had the second largest genome harbors maximum SSRs. Genome size may impact the frequency of SSRs, hence we have estimated the SSRs by taking 1 Mb length of each set of sequences analyzed as a reference. In this way, total relative abundance and total relative density were calculated. While maintaining the position of *A. niger* (256.4 and 1059.6) with the maximum frequency of SSRs, this promoted *A. terreus* (161.3 and 636.8) to the second place ahead of *A. oryzae* (140.8625 and 568.4367; Table 1). We further analyzed the percentage of different classes of repeats in their respective genomes. In *A. nidulans* and *A. terreus*, tri-nucleotide repeats constituted the maximum percentage of SSRs (38.25 and 42.15%) followed by tetra-nucleotide repeats (32.0 and 30.7%) while di-nucleotide repeats were the least (6.3 and 8.1%; Table 2). In *A. niger* and *A. oryzae*, tetra-nucleotide repeats (36.7 and 35.9%) were the most abundant repeats which were closely followed by tri-nucleotide repeats (35.7 and 30.1%), hexa-nucleotide repeats constituted the minimum number of repeats (7.5 and 10.0%). While comparing the most abundant motif, we observed that tri-nucleotide motif aag/ctt was the most favored motif in the genome of *A. nidulans*. Similarly, *A. terreus* genome showed preference for another tri-nucleotide repeat cgc/gcg (2.52%). Di-nucleotide repeat motif ga/tc (2.92%) was the most abundant motif in *A. niger* genome whereas *A. oryzae* preferred tetra-nucleotide repeat motif gaaa/tttc (2.64%; Table 3).

**Table 1:** Occurrence, relative abundance, and relative density of SSRs in the whole genome and transcript sequences of pathogenic and non-pathogenic *Aspergillus* species.

	***A. niger***		***A. terreus***		***A. nidulans***		***A. oryzae***	
	**WG**	**Transcripts**	**WG**	**Transcripts**	**WG**	**Transcripts**	**WG**	**Transcripts**
Size (Mb)	34.7	16.8	29.9	15.6	30.0	15.0	37.1	16.2
%GC	50.3	53.9	52.9	56.3	48.2	53.2	50.4	51.8
No. of SSR	8,896	935	4,823	742	2,919	540	5,226	550
Perfect SSR	8,024	899	4,526	725	2,824	527	4,963	524
	(90.2%)	(96.2%)	(93.84%)	(97.7%)	(96.74%)	(97.3%)	(94.96%)	(97.0%)
Compound SSR	864	36	285	17	90	14	256	26
	(9.7%)	(3.8%)	(5.9%)	(2.7%)	(3.08%)	(2.7%)	(4.8%)	(3.0%)
SSRs length (bp)	36,767	13,636	19,040	12,150	11,617	16,244	21,089	8,021
% length	0.11	0.06	0.06	0.06	0.03	0.12	0.05	0.05
RA (SSR/Mb)	256.4	55.6	161.3	47.5	97.3	36	140.8	33.3
RD (bp/Mb)	1,059.6	811.6	636.8	778.8	387.2	1,082	568.4	495.1

**Table 2:** Percentage, relative abundance, and relative density of SSRs in whole genome sequence sets of different species of *Aspergillus*.

	**Class**	**Count**	**Percentage (%)**	**RA**	**RD**
*A. niger*	Di	1,005	10.09	28.96	57.92
	Tri	3,561	35.74	102.62	307.87
	Tetra	3,660	36.73	105.48	421.90
	Penta	994	9.98	28.64	143.23
	Hexa	744	7.47	21.44	128.65
*A. terreus*	Di	416	8.07	13.91	27.83
	Tri	2,173	42.15	72.67	218.03
	Tetra	1,587	30.79	53.08	212.31
	Penta	533	10.34	17.83	89.13
	Hexa	446	8.65	14.92	89.49
*A. nidulans*	Di	191	6.31	6.37	12.73
	Tri	1,157	38.25	38.57	115.7
	Tetra	968	32	32.27	129.07
	Penta	362	11.97	12.07	60.33
	Hexa	347	11.47	11.57	69.4
*A. oryzae*	Di	588	10.65	15.85	31.698
	Tri	1,658	30.04	44.69	134.07
	Tetra	1,985	35.96	53.50	214.02
	Penta	735	13.32	19.81	99.06
	Hexa	554	10.04	14.93	89.59

**Table 3:** Most common repeat motif identified from perfect and compound microsatellite in the whole genome sequence of four *Aspergillus* species.

***A. niger***			***A. terreus***			***A. nidulans***			***A. oryzae***		
**Motif**	**Count**	**%**	**Motif**	**Count**	**%**	**Motif**	**Count**	**%**	**Motif**	**Count**	**%**
ga/tc	291	2.92	cgc/gcg	130	2.52	aag/ctt	78	2.58	gaaa/tttc	146	2.64
ag/ct	274	2.75	ccg/cgg	123	2.39	aga/tct	73	2.41	ga/tc	144	2.61
cag/ctg	236	2.37	ag/ct	120	2.33	gcatgc	70	2.31	aaag/cttt	132	2.39
cac/gag	200	2.01	aga/tct	118	2.29	cag/ctg	68	2.25	gaa/ttc	129	2.34
gca/tgc	188	1.89	ga/tc	114	2.21	ctc/gag	66	2.18	aag/ctt	126	2.28
agc/gct	187	1.88	gcc/ggc	113	2.19	atc/gat	59	1.95	at/at	122	2.21
cca/tgg	176	1.77	aat/att	105	2.04	agc/gct	58	1.92	ag/ct	120	2.17
acc/ggt	175	1.76	ctc/gag	105	2.04	at/at	58	1.92	aga/tct	96	1.74
tca/tga	164	1.65	cac/gtg	103	2.00	gaa/ttc	56	1.85	taca/tgta	95	1.72
aag/ctt	161	1.62	aag/ctt	99	1.92	tca/tga	56	1.85	aat/att	90	1.63
gaa/ttc	161	1.62	ata/tat	98	1.90	gga/tcc	45	1.49	ta/ta	86	1.56
aga/tct	158	1.59	gaa/ttc	97	1.88	ta/ta	44	1.45	aaaag/ctttt	83	1.50
ca/tg	146	1.47	cca/tgg	91	1.77	aatatt	43	1.42	agaa/ttct	83	1.50
atc/gat	143	1.44	gga/tcc	90	1.75	acc/cct	39	1.29	aaga/tctt	82	1.49
atg/cat	143	1.44	cga/tcg	87	1.69	ga/tc	39	1.29	ata/tat	75	1.36
act/agt	131	1.31	acc/ggt	84	1.63	gcc/ggc	36	1.19	taa/tta	72	1.30
ac/gt	125	1.25	gca/tgc	72	1.40	atg/cat	35	1.16	tca/tga	72	1.30
ctc/cag	122	1.22	cag/ctg	71	1.38	ata/tat	34	1.12	acc/ggt	70	1.27
gta/tac	122	1.22	at/at	70	1.36	cga/tcg	33	1.09	gaaaa/ttttc	70	1.27
cta/tag	115	1.15	taa/tta	62	1.20	taa	33	1.09	taaa/ttta	66	1.20
gga/tcc	114	1.14	ta/ta	61	1.18	aaag/cttt	31	1.02	aaat/attt	65	1.18
caa/ttg	113	1.13	tca/tga	57	1.11	aaga/tctt	31	1.02	cag/ctg	64	1.16
aat/att	98	0.98	agc/gct	54	1.05	ag/ct	31	1.02	cca/tgg	63	1.14
agg/cct	93	0.93	caa/ttg	54	1.05	cgc/gcg	30	0.99	gca/tgc	63	1.14
at/at	91	0.91	atc/gat	51	0.99	agaa/ttct	29	0.96	ctc/gag	61	1.11
aaag/cttt	90	0.90	atg/cat	50	0.97	cca/tgg	29	0.96	ac/gt	60	1.09
aca/tgt	85	0.85	gac/gtc	46	0.89	caa/tgg	28	0.93	atac/gtat	57	1.03
taca/tgta	82	0.82	agg/cct	45	0.87	agg/cct	27	0.89	atg/cat	56	1.01
atca/tgat	81	0.81	ctac/gtag	44	0.85	cac/gtg	27	0.89	aca/tgt	55	1.00
atac/gtat	79	0.79	aaga/tctt	40	0.78	ccg/cgg	26	0.86	gga/tcc	54	0.98

### Relative abundance and density of SSRs in the transcripts of *Aspergillus* spp.

In genic sequences, the maximum frequency of SSRs was observed in *A. niger* (935) transcripts which were followed by *A. terreus* (742) and *A. oryzae* (550). The relative abundance and relative density of SSRs also follow the same pattern as highest relative abundance and relative density of SSRs was observed in *A. niger* (55.6 and 811.0) while it was found lowest in *A. oryzae* (33.3 and 495.1; Table 1). While comparing the different classes of SSRs, we observed that the percentage of tri-nucleotide motifs were undoubtedly highest in all the transcripts. Hexa-nucleotide repeats were the second highest motifs in *A. nidulans* whereas in *A. niger, A. oryzae*, and *A. terreus* it was tetra-nucleotide repeats. Di-nucleotide motifs were the least abundant motifs in the transcripts (Supplementary Table 3). Overall, the frequency of SSR in the transcripts is much lower when compared to other Ascomycetes (Mahfooz et al., 2015, 2016). Analysis of most common repeats reveals preference of specific trinucleotide motifs in the transcripts of *Aspergillus* species. Motif aag/ctt was the most preferred motif in *A. nidulans* and *A. oryzae* however its percentage was significantly higher in *A. oryae* (6.60%) as compared to *A. nidulans* (5.22%). In remaining species, *A. niger* preferred cag/ctg motifs (5.30%) whereas *A. terreus* preferred ccg/cgg motifs (6.32%; Supplementary Table 4).

### Conservation of motifs among *Aspergillus* spp.

**Figure 1:**
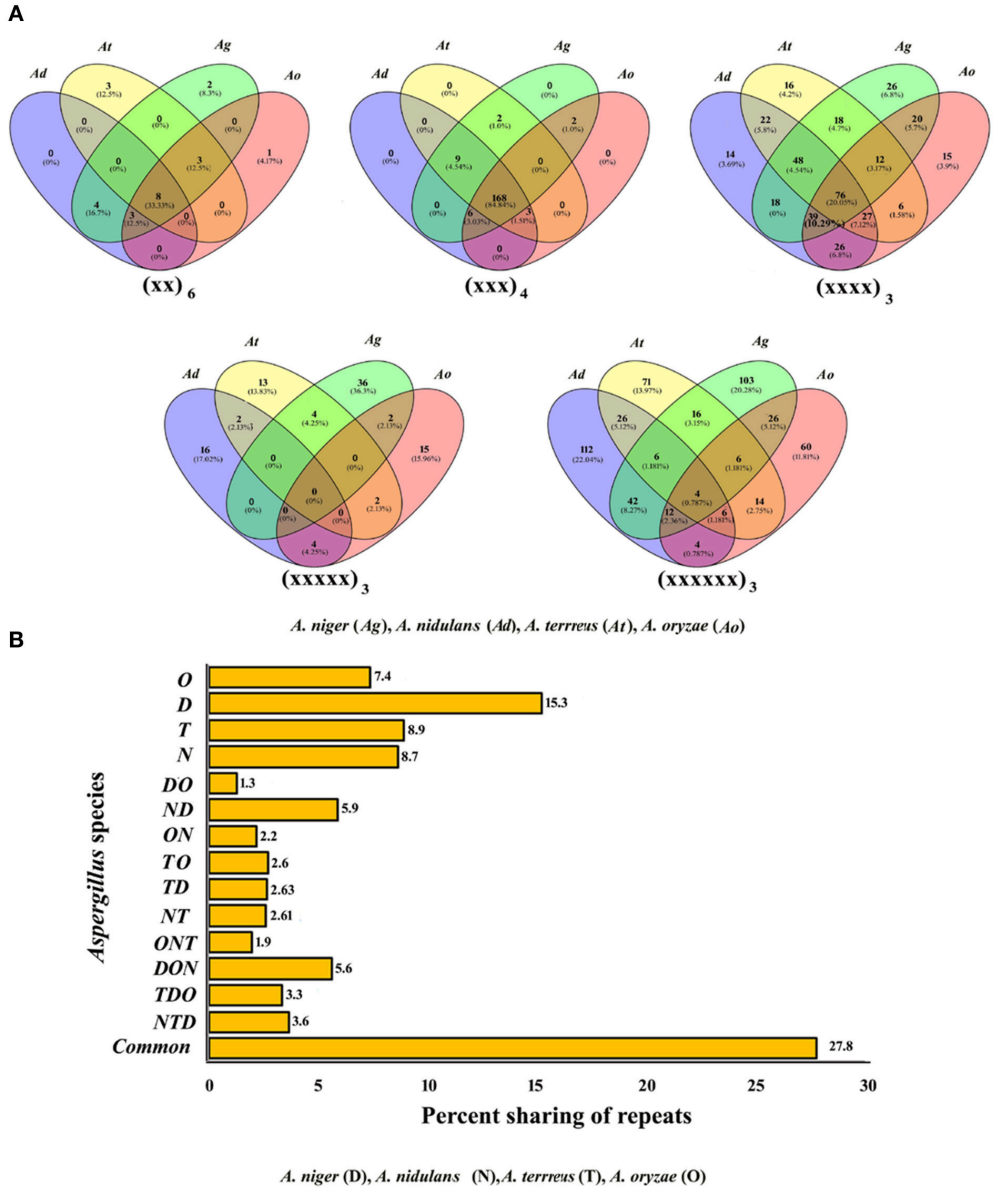
**(A)** Venn diagram showing sharing of different classes of motifs in the transcripts of *Aspergillus* species. **(B)** Graphical representation of sharing of all types of motifs in the transcripts of *Aspergillus*.

To analyze the developmental relationship among the *Aspergillus* species and to recognize unique motifs, every motif was examined for its counterpart in the transcripts of remaining species. The greatest number of motifs shared between all four transcripts was tri-nucleotide repeats (168, 84.8%) which were followed by di-nucleotide repeats (8, 33.3%), and tetra-nucleotide repeats (76, 20.1%; Figure 1A). Interestingly, none of the penta-nucleotide motifs were found to be shared within the transcripts of four species studied. Among the unique motifs, maximum unique motifs were observed as penta-nucleotide repeats in *A. nidulans* (Supplementary Table 5). While comparing the sharing of all classes of repeats among two species, it was noticed that *A. niger-A. nidulans* shared maximum percent of motifs (5.9%) whereas the least sharing was observed among *A. niger-A. oryzae* (1.3%). Among three species, it was the trio *A. nidulans–A. oryzae–A. niger* which showed the maximum conservation of motifs whereas least was observed within *A. oryzae–A. niger–A. terreus*. Maximum number of unique motifs was observed in *A. nidulans* (15.3%) which was followed by *A. terreus* (8.9%) and *A. niger* (8.7%), least was observed in *A. oryzae* (7.4%). A total of 27.8% of motifs were found conserved within all four species (Figure 1B).

**Figure 2:**
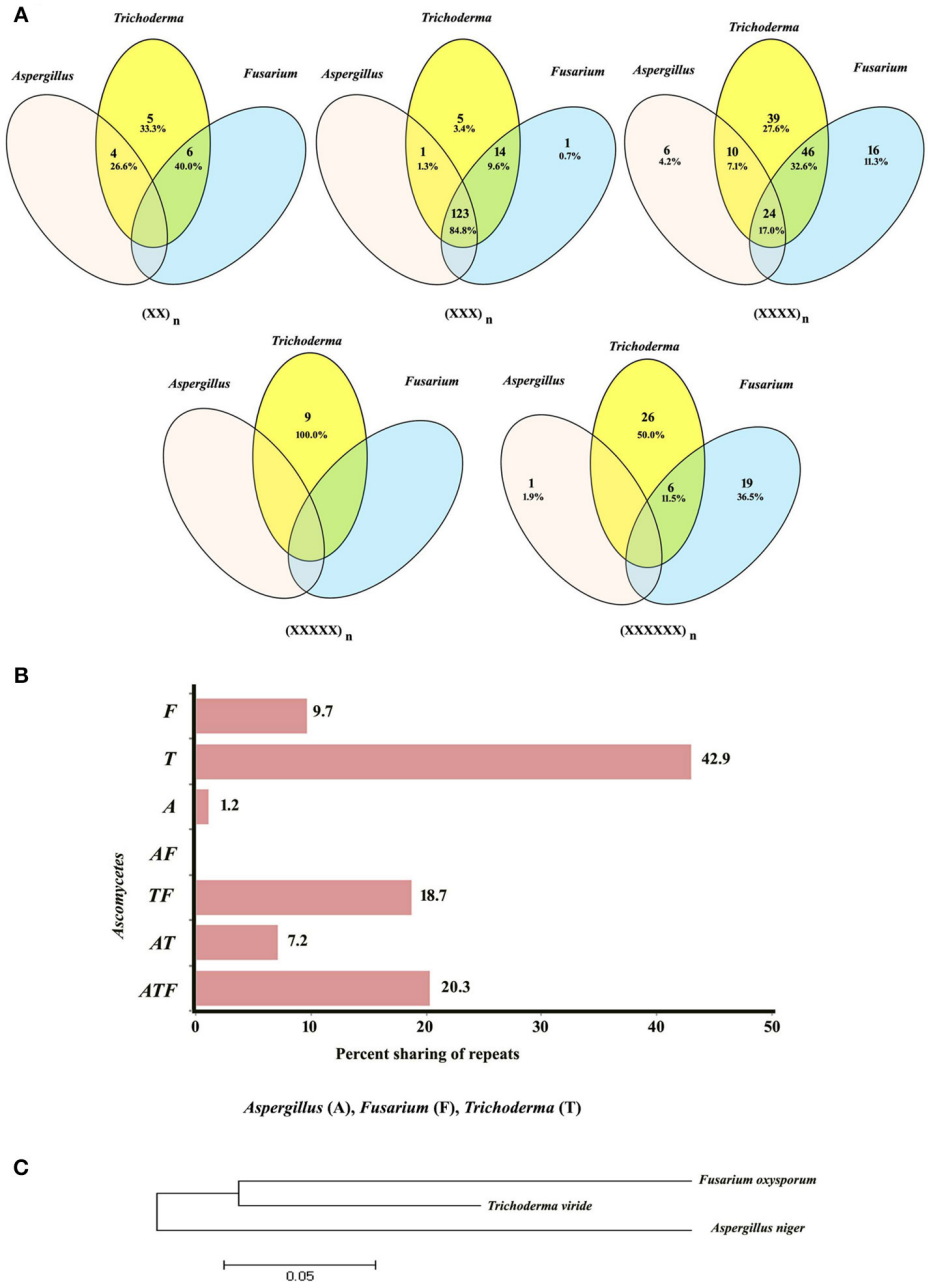
**(A)** Venn diagram showing sharing of different classes of motifs in the transcripts of Ascomycetes. **(B)** Graphical representation of sharing of all types of motifs in the transcripts of Ascomycetes. **(C)** Conserved gene (rRNA) based phylogeny.

Conservation of motifs was further analyzed at genus level among the members of Ascomycota. We have incorporated the most common motifs of *Fusarium* and *Trichoderma* from our previous analysis along with the common motifs identified in this study. As expected, tri-nucleotide repeats were the most common class of repeats shared between the three genera (Figure 2A), this was followed by tetra-nucleotide repeats. Interestingly, di-, penta-, and hexa-nucleotide repeats did not exhibit any conservation among the three genera. Further analysis of motif sharing among all classes revealed 20.3% conservation. Maximum conservation of motifs was witnessed among *Trichoderma* and *Fusarium* (18.7%) which was followed by *Aspergillus* and *Trichoderma* (7.2%). It is noteworthy that not a single motif was conserved within *Aspergillus* and *Fusarium* (Figure 2B). The maximum numbers of unique motifs were identified in *Trichoderma* while *Aspergillus* showed the least. In addition to the relationship we obtained through the conservation of motifs, we wanted to compare this relationship with the conserved region (18S) based phylogeny. To our surprise, both the methodologies resulted in an almost similar relationship within the Ascomycetes (Figure 2C).

### Codon usage

**Figure 3:**
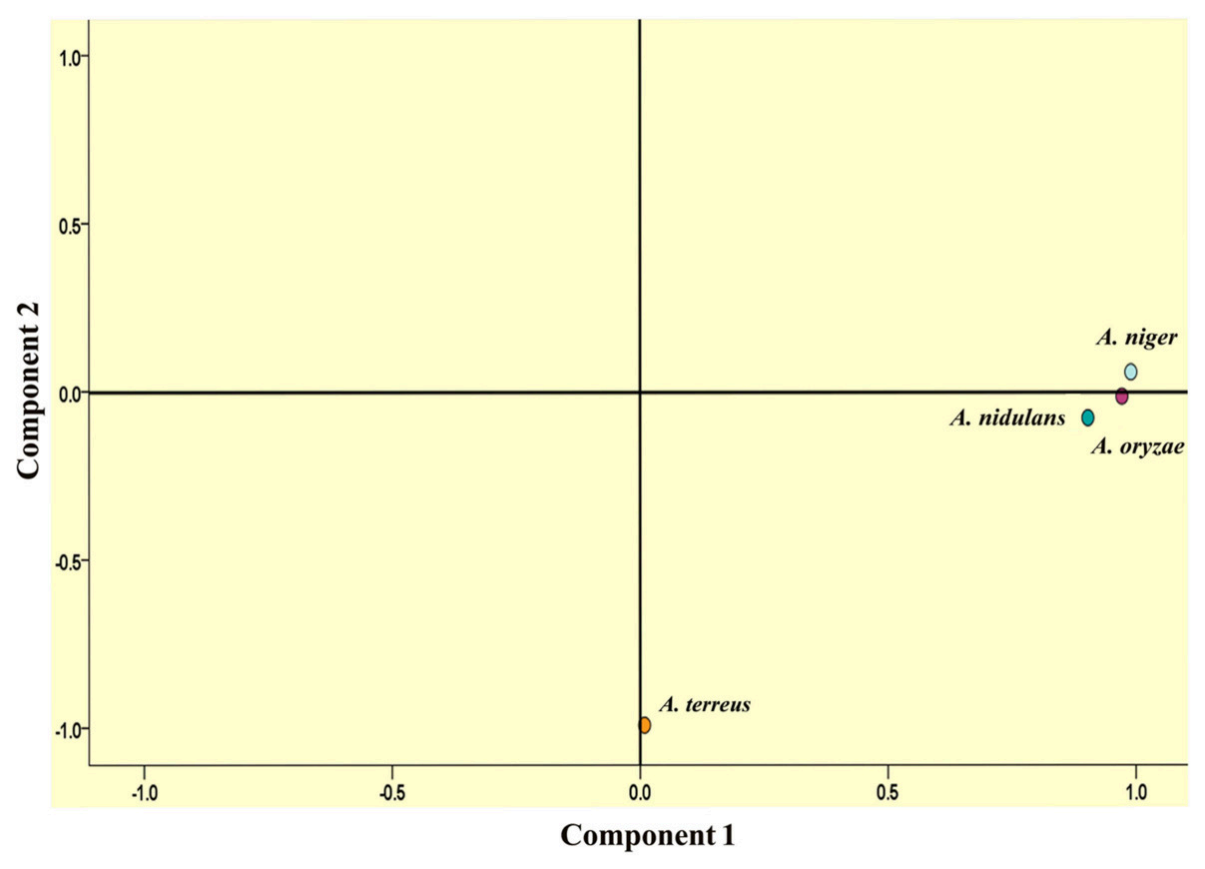
Distant clustering of *A. terreus* in PCA plot on the basis of amino acids encoded by tri-nucleotide SSRs in the transcript sequences.

Tri-nucleotide repeats in the transcripts have maximum chances of translation into protein. We analyzed all the trinucleotide repeats in order to get an insight of amino acids encoded by them. In *A. nidulans* and *A. terreus*, arginine coding motifs were in maximum whereas, in *A. niger*, glutamine coding motifs were in abundance. *A. oryzae* showed a preference for serine coding motifs. On the basis of amino acids encoded by different tri-nucleotide motifs, we intended to deduce a relationship among the four species. For this, we performed principal component analysis, a mathematical algorithm that reduces the dimensionality of the data while retaining most of the variation in the data set. The percentage of variance explained by the first component was 44.8% whereas it was 16.5% for the second (Figure 3). The PCA plot showed close clustering among *A. niger, A. nidulans*, and *A. oryzae*. The preference of amino acid is different in *A. terreus* as it clustered separately in the PCA plot.

### Diversity assessment among different isolates of *Aspergillus*

Out of 21, an aggregate of 12 SSR markers (six each from *A. niger* and *A. terreus*) amplified easily scorable amplicons running from 70 to 400 bp in all the isolates. Ten tri-nucleotide repeats and two di-nucleotide repeats were successfully amplified. Percentage polymorphism, number of alleles per locus and PIC value was utilized to demonstrate SSR polymorphism level. Among all the amplified markers, 10 markers (83.3%) were polymorphic, while rest two markers (16.6%) were monomorphic. A total of 22 alleles were amplified by 12 markers. We identified 1–4 alleles for every microsatellite locus with an average of 1.83 alleles for each marker. *A. niger* primers amplified 12 alleles with 2.0 alleles for every locus, while *A. terreus* primers amplified 10.0 alleles with 1.6 alleles for every locus. The highest number of alleles (4) were amplified by primer *At*539, while least of one allele was amplified with five markers viz. *An*868, *At*193, *At*257, *At*648, *At*660 (Table 4).

**Table 4:** Detail of locus, primer sequence, Tm, Motif, percentage polymorphism, No. of alleles, and PIC value of different primers used to evaluate genetic diversity within *Aspergillus* isolates.

	**Forward (5′–3′)**	**Reverse (5′–3′)**	**Tm °C**	**Motif**	**% poly-morphism**	**No. of alleles**	**PIC**
An23	tgtttcgagccagtttctat	gtagatgacttcgtcgatcc	54.8	(ct)_7_	100%	2	0.94
An172	ttatgctttcaacacatcca	gcaatctgatgagagaggtt	54.7	(gaa)_5_	100	2	0.97
An281	tcctctacaaaactctccca	gggaaagtggcgattg	55.3	(cta)_8_	100%	3	0.96
An868	caattcatatggaccctcag	ttggctttataacctcgtgt	55.2	(tg)_6_		1	0
An716	atcaaaatcgcaatctcatt	taatcgtagacggattcgag	55.1	(cat)_4_(cat)_4_(tca)_7_	100%	2	0.97
An517	acttgccatatagctccaga	caaccctgagactgaatagc	55.0	(ggc)_5_(ggc)_4_	100%	2	0.82
At527	gaagactacatctgttccgc	ttggtagaccaaactcatcc	54.9	(gac)6	100%	2	0.61
At193	atcgtcgcactcaatacaa	gggtctgaatacctctcctc	55.1	(cgt)4		1	0
At257	gattcctctggtcattggta	atgaagaacatgagacccag	54.9	(ctt)4	100%	1	0.91
At539	gaggccacaagatcacc	atatgaccgttggtacttgc	54.8	(ccg)5	100%	4	0.93
At648	ggatcgaagtttgaccact	caagaaaccaaagttggaag	55.0	(ggc)5	100%	1	0.85
At660	gtcgtctacctcaaggacag	atgtacaggatcgagcagaa	55.3	(gct)4	100%	1	0.24

The coefficient values between isolates extended from 0.28 to 1.0 with a mean of 0.62 for each of the 276 isolates combination utilized as a part of the present investigation. For microsatellite markers obtained from *A. niger*, the similarity coefficient value between isolates ranges from 0.54 to 1.00 with an average genetic diversity of 33.1%. Similarly, with *A. terreus* SSR markers, the similarity coefficients between isolates ranges from 0.69 to 1.00 with 34.5% genetic diversity (Table 5).

**Table 5:** A comparison between *A. niger* and *A. terreus* markers in order to estimate the level of polymorphism revealed by them.

	***A. niger***	***A. terreus***	**Total**
Markers used	10	10	20
Marker amplified	6	6	12
No. of polymorphic markers	5 (83.3%)	5 (83.3%)	10
No. of monomorphic markers	1 (16.6%)	1 (16.6%)	2
Average PIC value	0.77	0.58	0.68
No. of alleles amplified	12	10	22
Similarity coefficient value (Avg.)	0.54	0.69	0.62

**Figure 4:**
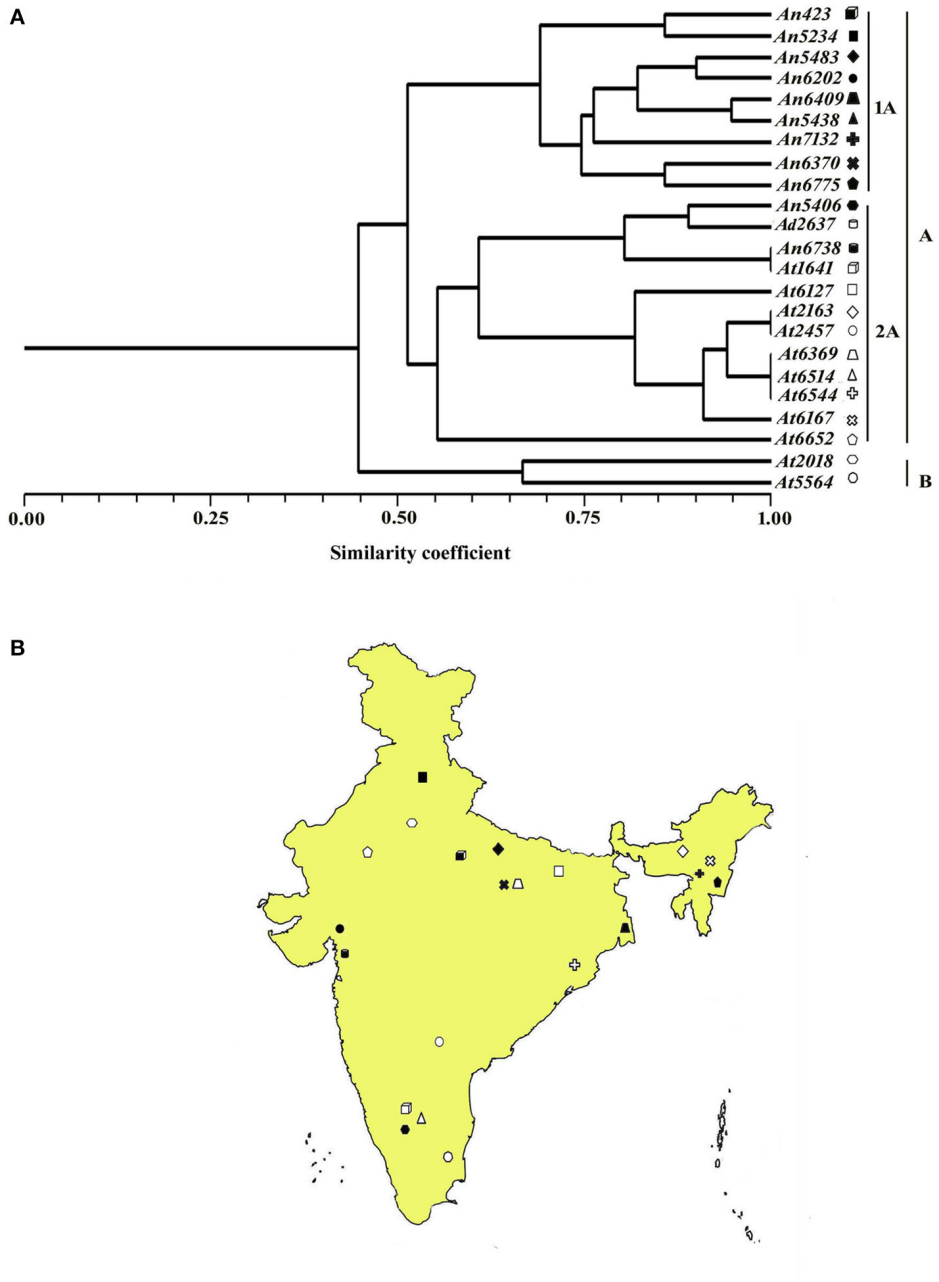
**(A)** Dendrogram showing genetic relationship among the *Aspergillus* isolates based on 12 microsatellite markers. Scale indicates Jaccard's coefficient of similarity. A and B indicates main clusters. 1A, 2A, indicate sub-clusters within main cluster A. **(B)** Map of India showing the geographical location of different isolates used for diversity analysis in this study.

The most elevated similarity coefficient (1.0) was seen between *A. terreus* isolates *At*2167-*At*2457, *At*6369-*At*6514, *At*6544-*At*6369, and *At*6514-*At*6544 whereas the most diverse (similarity coefficient value 0.29) isolates were *An*423 and *At*5564. The dendrogram constructed based on similarity index resulted in two main clusters A and B. Most of the isolates from *A. niger, A. nidulans*, and *A. terreus* grouped together in cluster A whereas cluster B contained only *A. niger* isolates. Cluster A was further subdivided into subgroups 1A and 2A. 1A comprised exclusively of *A. niger* isolates, whereas 2A contained a majority of *A. terreus* isolates (Figures 4A,B).

## Discussion

The members of genus *Aspergillus* is having the reputation of being the most diverse. It has been reported that the most closely related species are as divergent as human and mice (Machida et al., 2005; Fedorova et al., 2008). This divergence is evident in the large variation among the frequency of microsatellite obtained in our study as well. The occurrence of significantly higher frequency of SSR in *A. niger* was surprising. In earlier studies, it has been reported that the frequency of SSRs is positively correlated with the G+C content of the genome (Tian et al., 2011; Mahfooz et al., 2016) however this is not true for *Aspergillus* where no such correlation was observed. This uneven frequency of SSR distribution was also observed among the species of *Drosophylla* as well (Ross et al., 2003). The most probable reason for the higher SSR frequency in *A. niger* is the presence of a large number of tetra- and di-nucleotide repeats in the whole genome, however, this also fall short in explaining the higher frequency of SSRs in *A. niger*. We further analyzed the frequency of SSR in the transcripts of *Aspergillus* species where the frequency of SSRs was found to be positively correlated with the G+C content of transcripts. Although, we obtained a weak correlation value (*r*^2^ = 0.247), this might explain the difference in frequency of SSRs among the transcripts. We further noticed a significantly lower frequency of SSRs in the transcripts of *A. oryzae* as compared to other species. This was interesting as *A. oryzae* has been reported to display two-fold higher rate of insertion which is in parallel with its largest genome size (Machida et al., 2005). The most potential explanation of lower frequency of SSRs in the *A. oryzae* transcript is the acquisition of lineage-specific sequences, since we are estimating the frequency of SSR per Mb of transcripts, the presence of extra sequences might have diluted the frequency of SSRs. It is evident from the results that pathogenic species of *Aspergillus* contained more repeats as compared to the non-pathogenic one in both whole genomes as well as in the transcripts. It has been reported that in pathogens, SSRs can improve antigenic fluctuation of the pathogen population in a procedure that balances the host immune response (Mrazek et al., 2007).

Tri-nucleotide SSRs were unanimously the most abundant class of SSRs in the transcripts of *Aspergillus* species. The higher abundance of tri-nucleotide SSRs in the transcripts is expected as any expansion or contraction within these repeats did not disturb the reading frame, hence these repeats are well-tolerated in the coding region (Katti et al., 2001; Garnica et al., 2006). The higher occurrence of aag/ctt repeats in *A. nidulans* and *A. oryzae* was expected as it has been reported that due to positive selection, aag repeats are predominant in 5′ flanks close to those genes whose products are preferentially involved in transcription (Zhang et al., 2004). Our previous analysis in *Fusarium* also reveals the predominance of these repeats among its three species (Mahfooz et al., 2015). Since cag codes for glutamine, its abundance in the transcripts of *A. niger* might be attributed to its reputation of being a polar zipper protein-protein interaction domain (Michelitsch and Weissman, 2000).

We further analyzed the conservation of motifs among *Aspergillus* species, which resulted in a low conservation (27.8%) was obtained when compared to other Ascomycetes (Mahfooz et al., 2015, 2016), this again reflected its diverse genome architecture (Rokas et al., 2007; Fedorova et al., 2008). Among the three species, maximum conservation was obtained in the trio *A. niger*–*A. nidulans*–*A. oryzae* (5.3%) which may be explained on the basis of sequence conservation within 5,000 non-coding regions with the abundance of repeats actively conserved within these species (Galagan et al., 2005). It has been reported that of 8,695 genes in *A. niger*, 78% showed conservation of neighboring orthologs in at least one species (Pel et al., 2007). This might be the reason why *A. niger* and *A. nidulans* showed maximum motif sharing among themselves. Our previous analysis of motifs conservation in *Fusarium* and *Trichoderma* prompted us to analyze it at the genus level. The three genus shared only 20.3% common motifs despite the fact that 80% of genes in *Aspergillus* have homologs in other lineages of fungi (de Vries et al., 2017). Higher conservation of motifs among *Fusarium* and *Trichoderma* (18.7%) was anticipated as *Trichoderma* and *Fusarium* belongs to Sordariomycetes whereas *Aspergillus* falls under Eurotiomyceties (Grigoriev et al., 2014). The least number of unique motifs obtained in *Aspergillus* suggests a low level of genetic heterogeneity in *Aspergillus* as compared to other Ascomycetes. It was thought provoking to witness similar relationship on the basis of hyper-variable and conserved regions. The possible explanation for this might be attributed to the fact that within the genes, apart from long stretches of nucleotides, short stretches are also conserved with a possibility of change in a number of repeats.

Due to positive selection, changes in amino acids has been witnessed in domesticated fungi probably because of the strong selection pressure exerted by humans. The genetic code itself can also provide unexpected adaptive amino acid changes. In *Candida albicans*, incorporated serine residues were witnessed at sites where leucine was previously placed and this replacement was well-tolerated in the genome (Miranda et al., 2013). This might be one of the reasons why *A. terreus* distantly clustered in the PCA plot. Apart from this, higher abundance of arginine, alanine, and proline coding repeats might also be responsible for the distant clustering of *A. terreus* in PCA plot. The acquisition of additional repeats in proteins of *A. terreus* may help to fine tune its function and/or modify some of its properties (Mularoni et al., 2010).

The primers designed in the present study were further validated for its ability to detect polymorphism. Till date, only six polymorphic microsatellites were developed for *A. niger* (Esteban et al., 2005) which were insufficient for estimating genetic diversity. Earlier RAPD markers were widely used for genetic characterization of *Aspergillus* isolates. Diversity and phylogenetic relationship of 12 *Aspergillus* species isolated from Tehran were studied using 11 RAPD markers (Kermani et al., 2016). The authors obtained similarity coefficient ranged from 0.02 to 0.40 indicating a wide diversity within *Aspergillus* isolates. Higher genetic diversity was also obtained in *A. terreus* isolates collected from Houston, Texas, and Innsbruck using RAPD markers (Lass-Florl et al., 2007). A higher range of similarity coefficient obtained in our study with SSR markers might be attributed to the fact that in our analysis only three species were analyzed. The newly develop markers in *A. niger* and *A. terreus* along with previously published marker in *A. niger* revealed that for the distinction of a broad range of *A. niger* and *A. terreus* strains and to analyze intraspecies variation among them, these markers are sufficient. The available markers can address issues such as pathogenicity, ecology, and species differentiation within the genus *Aspergillus*. In addition to this, the unique motifs obtained in this study may be utilized for the development of species-specific markers.

## Author contributions

Conceived and designed the experiments: SM, AM. Performed the experiments: SM, SS, NM. Analyzed the data: SM. Wrote the manuscript: SM, AM.

### Conflict of interest statement

The authors declare that the research was conducted in the absence of any commercial or financial relationships that could be construed as a potential conflict of interest.

## Supplementary material

Supplementary Table 1 Primers to amplify motifs in different *Aspergillus* species.

Supplementary Table 2 Accession number, name of species source of isolation and place of collection of different *Aspergillus* isolates used in the present study.

Supplementary Table 3 Percentage, relative abundance, and relative density of SSRs in transcripts of different species of *Aspergillus*.

Supplementary Table 4 Most common repeat motif identified from perfect and compound microsatellite in the transcripts genome sequence of four *Aspergillus* species.

Supplementary Table 5 A list of unique motifs obtained in the transcript sequences of four *Aspergillus* species analyzed.
