# Supplementary material for: A Comparison of Microsatellites in Phytopathogenic Aspergillus Species in Order to Develop Markers for the Assessment of Genetic Diversity among Its Isolates
Source: Front Microbiol. 2017 Sep 20;8:1774. doi: 10.3389/fmicb.2017.01774 (PMC5611378; doi:10.3389/fmicb.2017.01774)
Supplement: Supplementary Table 2 — Accession number, name of species source of isolation and place of collection of different Aspergillus isolates used in the present study. [file Table2.DOCX]

|  |  |  |  |  |
| --- | --- | --- | --- | --- |

**Supplementary table S2: Accession number, name of species source of isolation and place of collection of different *Aspergillus* isolates used in the present study**

| **S. No** | **Accession number** | **Species** | **Source** | **Place** |
| --- | --- | --- | --- | --- |
| 111 1 | 321 | *A.niger* | Contaminated yeast culture | New Delhi |
| 2 | 423 | *A.niger* | Damaged wire | Kanpur |
| 3 | 5231 | *A.niger* | Waste of sugarcane Industries | Chandigarh |
| 4 | 5406 | *A.niger* | Sugarcane | Coimbatore |
| 5 | 5483 | *A.niger* | Sugarcane mill effluent | Saharanpur |
| 6 | 6202 | *A.niger* | *Citrus reticulate* | Anand |
| 7 | 6370 | *A.niger* | *Sesbania aculeata* | Varanasi |
| 8 | 6409 | *A.niger* | Soil of Jute | Calcutta |
| 9 | 6438 | *A.niger* | Groundnut rhizosphere | Bikanare |
| 10 | 6738 | *A.niger* | Sorghum seeds | Navsari |
| 11 | 7132 | *A.niger* | Tea soil | Silchar |
| 12 | 6775 | *A.niger* | Groundnut | Imphal |
| 13 | 1641 | *A.terreus* | Soil | Mysore |
| 14 | 2018 | *A.terreus* | Wheat grains | New Delhi |
| 15 | 2163 | *A.terreus* | Soil | Assam |
| 16 | 2457 | *A.terreus* | Potato | Hyderabad |
| 17 | 5564 | *A.terreus* | Dyeing industry effluent | Tamilnadu |
| 18 | 6127 | *A.terreus* | *Agaricus bitorquis* | Bihar |
| 19 | 6369 | *A.terreus* | *Sesbania aculeata* | Varanasi |
| 20 | 6514 | *A.terreus* | Potato | Bangalore |
| 21 | 6544 | *A.terreus* | Coconut rhizosphere | Dapoli |
| 22 | 6167 | *A.terreus* | White fly | Medziphama |
| 23 | 2637 | *A.nidulans* | Soil | New Delhi |
|  |  |  |  |  |
