# Supplementary material for: A Comparison of Microsatellites in Phytopathogenic Aspergillus Species in Order to Develop Markers for the Assessment of Genetic Diversity among Its Isolates
Source: Front Microbiol. 2017 Sep 20;8:1774. doi: 10.3389/fmicb.2017.01774 (PMC5611378; doi:10.3389/fmicb.2017.01774)
Supplement: Supplementary Table 3 — Percentage, relative abundance, and relative density of SSRs in transcripts of different species of Aspergillus. [file Table3.DOCX]

**Supplementary table S3: Percentage, relative abundance, and relative density of SSRs in transcripts of different species of *Aspergillus***

|  | **Class** | **Count** | **Percentage** | **RA** | **RD** |
| --- | --- | --- | --- | --- | --- |
| *A. nidulans* | **Di** | 14 | 2.52% | 0.93 | 1.87 |
|  | **Tri** | 295 | 53.06% | 19.67 | 59 |
|  | **Tetra** | 110 | 19.78% | 7.33 | 29.33 |
|  | **Penta** | 17 | 3.06% | 1.13 | 5.67 |
|  | **Hexa** | 120 | 21.58% | 8 | 48 |
| *A. niger* | **Di** | 22 | 2.47% | 1.31 | 2.62 |
|  | **Tri** | 456 | 51.12% | 27.14 | 81.43 |
|  | **Tetra** | 194 | 21.75% | 11.55 | 46.19 |
|  | **Penta** | 47 | 5.27% | 2.8 | 13.99 |
|  | **Hexa** | 173 | 19.39% | 10.3 | 61.78 |
| *A. oryzae* | **Di** | 11 | 1.91% | 0.68 | 1.36 |
|  | **Tri** | 329 | 57.12% | 20.30 | 60.92 |
|  | **Tetra** | 122 | 21.18% | 7.53 | 30.12 |
|  | **Penta** | 19 | 3.3% | 1.17 | 5.86 |
|  | **Hexa** | 95 | 16.49% | 5.86 | 35.18 |
| *A. terreus* | **Di** | 18 | 2.37% | 1.15 | 2.31 |
|  | **Tri** | 464 | 61.13% | 29.74 | 89.23 |
|  | **Tetra** | 142 | 18.71% | 9.10 | 36.41 |
|  | **Penta** | 20 | 2.64% | 1.282051 | 6.41 |
|  | **Hexa** | 115 | 15.15% | 7.37 | 44.23 |
