# Supplementary material for: A Comparison of Microsatellites in Phytopathogenic Aspergillus Species in Order to Develop Markers for the Assessment of Genetic Diversity among Its Isolates
Source: Front Microbiol. 2017 Sep 20;8:1774. doi: 10.3389/fmicb.2017.01774 (PMC5611378; doi:10.3389/fmicb.2017.01774)
Supplement: Supplementary Table 4 — Most common repeat motif identified from perfect and compound microsatellite in the transcripts genome sequence of four Aspergillus species. [file Table4.DOCX]

**Supplementary table S4: Most common repeat motif identified from perfect and compound microsatellite in the transcripts genome sequence of four *Aspergillus* species**

| ***A. nidulans*** | | | ***A. niger*** | | | ***A. oryzae*** | | | ***A. terreus*** | | |
| --- | --- | --- | --- | --- | --- | --- | --- | --- | --- | --- | --- |
| motif | count | % | motif | count | % | motif | count | % | motif | count | % |
| aag/ctt | 29 | 5.22 | cag/ctg | 52 | 5.30 | aag/ctt | 38 | 6.60 | ccg/cgg | 48 | 6.32 |
| aga/tct | 25 | 4.50 | aag/ctt | 33 | 3.36 | cag/ctg | 24 | 4.17 | gcc/ggc | 45 | 5.93 |
| gaa/ttc | 21 | 3.78 | cac/gtg | 32 | 3.26 | ctc/gag | 24 | 4.17 | cgc/gcg | 41 | 5.40 |
| cag/ctg | 19 | 3.42 | cca/tgg | 32 | 3.26 | agatct | 23 | 3.99 | ctc/gag | 36 | 4.74 |
| ctc/gag | 19 | 3.42 | gaa/ttc | 32 | 3.26 | gaa/ttc | 23 | 3.99 | cga/tcg | 32 | 4.22 |
| gca/tgc | 16 | 2.88 | ctc/gag | 30 | 3.05 | acc/ggt | 18 | 3.13 | cca/tgg | 25 | 3.29 |
| agc/gct | 14 | 2.52 | gca/tgc | 30 | 3.05 | gga/tcc | 18 | 3.13 | cag/ctg | 24 | 3.16 |
| cgc/gcg | 14 | 2.52 | agc/gct | 27 | 2.75 | tca/tga | 18 | 3.13 | aag/ctt | 23 | 3.03 |
| ccg/cgg | 13 | 2.34 | atg/cat | 25 | 2.55 | caa/ttg | 15 | 2.60 | acc/ggt | 22 | 2.90 |
| gcc/ggc | 13 | 2.34 | aga/tct | 24 | 2.44 | gca/tgc | 15 | 2.60 | gaa/ttc | 18 | 2.37 |
| gga/tcc | 12 | 2.16 | caa/ttg | 22 | 2.24 | agc/gct | 14 | 2.43 | gac/gtc | 18 | 2.37 |
| tca/tga | 11 | 1.98 | gga/tcc | 22 | 2.24 | cca/tgg | 13 | 2.26 | agc/gct | 17 | 2.24 |
| cga/tcg | 10 | 1.80 | atc/gat | 18 | 1.83 | gat/atc | 11 | 1.91 | gga/tcc | 17 | 2.24 |
| gac/gtc | 10 | 1.80 | gcc/ggc | 18 | 1.83 | cac/gtg | 10 | 1.74 | gca/tgc | 15 | 1.98 |
| acc/ggt | 9 | 1.62 | tca/tga | 18 | 1.83 | cgc/gcg | 10 | 1.74 | aga/tct | 13 | 1.71 |
| cac/gag | 9 | 1.62 | acc/ggt | 17 | 1.73 | gcc/ggc | 9 | 1.56 | caa/ttg | 13 | 1.71 |
| cca/tgg | 9 | 1.62 | aac/gtt | 15 | 1.53 | act/agt | 8 | 1.39 | cac/gtg | 13 | 1.71 |
| agg.cct | 8 | 1.44 | aca/tgt | 15 | 1.53 | ccg/ccg | 8 | 1.39 | acg/cgt | 12 | 1.58 |
| atc/gat | 8 | 1.44 | agg/cct | 13 | 1.32 | cga/tcg | 8 | 1.39 | cg/cg | 8 | 1.05 |
| atg/cat | 7 | 1.26 | ccg/cgg | 13 | 1.32 | aca/tgt | 7 | 1.22 | agg/cct | 7 | 0.92 |
| ga/tc | 7 | 1.26 | ga/tc | 12 | 1.22 | agg/cct | 6 | 1.04 | ctcc/ggag | 7 | 0.92 |
| aca/tgt | 6 | 1.08 | acg/cgt | 10 | 1.02 | catc/gatg | 6 | 1.04 | gcga/tcgc | 7 | 0.92 |
| acg/cgt | 6 | 1.08 | cctc/gagg | 10 | 1.02 | ga/tc | 6 | 1.04 | atg/cat | 6 | 0.79 |
| caa/ttg | 6 | 1.08 | gac/gtc | 10 | 1.02 | cttc/gaag | 5 | 0.87 | ccgt/acgg | 6 | 0.79 |
| ag/ct | 5 | 0.90 | cga/tcg | 9 | 0.92 | gaaa/tttc | 5 | 0.87 | cggc/gccg | 6 | 0.79 |
| gaagag/ctcttc | 5 | 0.90 | cagc/gctg | 8 | 0.81 | aaag/cttt | 4 | 0.69 | gat/atc | 6 | 0.79 |
| aagc/gctt | 4 | 0.72 | cgc/gcg | 8 | 0.81 | agaa/ttct | 4 | 0.69 | aac/gtt | 5 | 0.66 |
| caag/cttg | 4 | 0.72 | act/agt | 6 | 0.61 | atgg/ccat | 4 | 0.69 | ccag/ctgg | 5 | 0.66 |
| ctcc/ggag | 4 | 0.72 | agca/tgct | 6 | 0.61 | cagc/gctg | 4 | 0.69 | tca/tga | 5 | 0.66 |
| tcca/tggt | 4 | 0.72 | aggg/ccct | 6 | 0.61 | gaggat/atcctc | 4 | 0.69 | agcg/cgct | 4 | 0.53 |
