# Supplementary material for: A Comparison of Microsatellites in Phytopathogenic Aspergillus Species in Order to Develop Markers for the Assessment of Genetic Diversity among Its Isolates
Source: Front Microbiol. 2017 Sep 20;8:1774. doi: 10.3389/fmicb.2017.01774 (PMC5611378; doi:10.3389/fmicb.2017.01774)
Supplement: Supplementary Table 5 — A list of unique motifs obtained in the transcript sequences of four Aspergillus species analyzed. [file Table5.DOCX]

**Supplementary table S5: A list of unique motifs obtained in the transcript sequences of four *Aspergillusa* species analyzed**

|  | *A. niger* | *A. terreus* | *A. nidulans* | *A. oryzae* |
| --- | --- | --- | --- | --- |
| di | ta, tg | cg, gc, gt |  | at |
| tri | aat ,att , gta ,gtt ,tat |  | taa | act, agt |
| tetra | acat, aggg, atac, atct, caaa, cacg, cagg, cata ccac, ctat, ctgt, gaat,gagg, gcta, ggga, ggtg, taat, tagc,tatc, tatg, tgac, tgat, tgta, tgtt,ttag,tttc | agct, aggc, tgc, cagt, gacg, gatc, gcaa, gcgg, ggcg, gtcc, gtcg, tacc, tcat, tccg, tcctc, tctg | acgc, actt, agtc, atcg, caat, catc, cctt, cgca, cggg, gcag, gggt, gttc, gttt ,tatt | aaac, aact,aagg, acaa,acag,actc,  atca,atgt,cacc, ccaa,cgtt,gaag,  gcct,tgcg,ttgg |
| penta | atagg,atgtg,caagg,cacac,cacct,cagcg,cccct,ccttt,cgaca,cgatt, ctgga, ctttt, gaggg, gatac, gatgt, gcaag, gccca, ggaat, gggga, gtttt, taacc, tatta, tccag, tcccg, tccgc, tctag,tctat, tctcc, tgaat, tgatg, tgggc, ttctt, ttgca, ttcc, ttttc, | aagac, agaca,cccat,ccgac, cctcg,cgcgc, cggat,gcagc,gcccc,  gccga,gcgcg,gggag, tgcag | acgct, agacg,atgga, caagt,cagag, cgagg, cgatc, cgctc, gaaac, gattg, gcaaa, gggaa, ggtct, tcccc, tgcga, ttcgg | agaag, atcca, cacat, ccaac,  ccctc, ccggt, ctcca,ctctc,  gaaaa,gaggc, tattt,tccaa,  tcctc,tcttt, ttgtc |
| hexa | aaaagg,aaacca,aaagaa,aaagga,aaaggg,aacact,  aacagc,aaccag,aacgag,aacgcc,aacggg,aagaac,  aagacg,aatcca,acaacc,acaagg,acacaa,acaccc,acacgc,acagct,accaac, accaca, acccca, acgaag, acgatg, actacc, actgcc, actgga, agaaac, agccaa, agcccc, agcgaa, agggat, ataacc,atcaac,atgagg,atgtga,caaagg,caaatc,  caacac,caacag,caacct,caatgc,cacatc,cagcca,caggat,caggct,cagggt,catcct, catgat, ccaaca, ccaacc, ccaagt, cccgag, ccgcag, ccggct, ccgtct, cgtgac, cctgcg, cctgct, cggctc, cggtaa, cggtgc,ctcaag,ctgcct,ggggat,  gggtga,ctccgg,ctctgg,ctgccc,gaggct,gagggc,gatcgc,gattcc,gcaagg, gcagcc, gccaca, gccgaa, gcggca, gcgtcg, gctcag,gctcct,gctcga,ggacat,  ggagca,ggcgag,ggctac,ggcttc,gggacg,gtgctg,tcacga,tcactg,tccacc, tccctc, tctact, tctttt, tgagaa, tgagcc, tgctcc, tggaag, tggcac,tggcat,  tgtgct | aaccac, accaag,accatg, acgcag, actggc, aggccg, agtcga, agtggc, atcgcg, atctat,atgcta,caacca, caacgc, caccag,cacctt, cacgaa, cagacc, ccacaa, ccacgg, ccctct, ccgacg, ccggag,ccgtcg,cctagt, cctccg, cctctt,cgaacc, cgagtc, cgcaac, cgcaga, cgcgtc,cgctcc,cggaca,  cggacg,cgggat,ctcggg, ctctac, gacctc,gacgcc, gagaaa, gcacca, gccaaa, gccgac, gccgct,gcctcg, gcgcag, gcgccg,gcggcc, gcggct, gctctg, gctgcg, ggactg, ggcaag, ggcagc,ggcgcc, gggact,gggcga,  gtcgcg,gtgaag,tcagca,tcctcg, tcgccc, tctcac,tgaagt, tgagaa, tgagag,  tgcggc,tggaac,tggatg, ttctct,ttctga | aaaaat, aacaag,aagctg,aagggc, aagtcc, acgagg,acgtct,agacga,  agacgg,agagga,agcgat,agcggc, aggaag,aggaga,aggtcg,agtccc,  ataccc, atcggc,atgcaa, caacat, caagcc,caagga, cacaat, cacctc,  cagcct,cagcga, cagtcg,ccaatc, ccagag, ccagct, ccagtg, cccgaa,  ccctca,ccctcg, ccctgc, ccgaga, ccgagt, ccgcct,ccgcga, cctcag,  cctcat, cctcgg, cctgcc,cgaaga, cgacaa, cgacag,cgacgc, cgcagc,  cgccag, cgctgc, cggact, cgtgag,ctaagg,ctcagt,ctccac, ctggtg,  cttcag, cttcca,gaacaa,gaacgg, gacaat,gaccag,ggtcag,ggtctg,  ggtgcc,ggtgct,ggttcg,cgagaa, ctcgcc,ctgcat,ctgccc,gacgaa,  gacggg, gactcc, gactcg, gagagc, gagagt, gagcag,gagccc,gatgac,  gatgca,gcaatc,gcactg,gcagat, gcagcg,gcaggg,gccctc,gccgcg,  gcctgc,gcgacc, gcgcca,gcgcct, gctccg,ggactc,ggcgca,ggctca,  ggctga,gggaat,gggcac,gtctgc, tcaagg,tcagag,tcccgc,tccggc,  tcggat,tctgaa,tgagac,tgcaac, tggctc,tgggca, tggggc,ttcggc | aacctc,aagccg, aatacc,acaatc,  acgacc,acttgg, agctct,aggaca,  agggtc,atgacg, atgctg,atggtc,  caatcc,cacggc, catcaa,cccact,  ccccct,ccccgg, cgatg,ccgccc,  ccgtgg,cctctg, cgagga,cgcctc,  cgtgat,ctactg, cttcta,gaaccc,  gacaac,gacgca, actac,gagcac,  gatgtt,gcagaa, gccatt,gccgca,  gcgcgg,gctccc, ctgca,ggaagc,  ggacag,ggagcc, gcaat,ggctcg,  ggtccc,gtggca, gttcct,tcaaca,  tcacca,tcagga, tcattc,tcgcag,  tcgggc,tctggc, tcttca,tgaaga,  tgacga,tgcaga, tgccac,tttctt |
